# Chronic exposure to high altitude and the presence of coronary ectasia in patients with ST elevation myocardial infarction

**DOI:** 10.47487/apcyccv.v4i4.329

**Published:** 2023-12-27

**Authors:** Manuel Chacón-Diaz

**Affiliations:** 1 Instituto Nacional Cardiovascular INCOR, EsSalud, Lima, Perú. Instituto Nacional Cardiovascular INCOR, EsSalud Lima Perú; 2 Universidad Particular Cayetano Heredia, Lima, Perú. Universidad Peruana Cayetano Heredia Universidad Particular Cayetano Heredia Lima Peru

**Keywords:** Coronary artery disease, Ectasia, Altitude, Myocardial Infarction, Enfermedad de la Arteria Coronaria, Ectasia, Altitud, Infarto de Miocardio

## Abstract

**Objective.:**

To evaluate the association between chronic exposure to high altitude and the presence of coronary ectasia (CE) in patients with ST-segment elevation myocardial infarction (STEMI) treated in a highly specialized cardiovascular reference hospital in Peru.

**Materials and methods.:**

Retrospective matched case-control study. The cases were patients with CE and controls without CE. The relationship between CE and chronic exposure to high altitude was evaluated considering intervening variables such as arterial hypertension, diabetes mellitus, dyslipidemia, smoking, and hematocrit values. Patients with chronic inflammatory pathologies, chronic obstructive pulmonary disease, and previous revascularization were excluded. Multivariate logistic regression was applied to obtain the OR value and their respective confidence intervals.

**Results.:**

Eighteen cases and 18 controls were studied, most of them were men with an average age of 65 years. Thirty-six percent of the population came from high altitude; in this group 76.9% had coronary ectasia of the infarct-related artery. The mean hematocrit value was slightly higher in the high-altitude native (46 ± 7% versus 42 ± 5%, p=0.094). Multivariate conditional logistic regression did not find a significant relationship between exposure to high altitude and the risk of presenting CE (OR:6.03, IC95%: 0.30-118, p=0.236).

**Conclusions.:**

In patients with STEMI, we found no association between chronic exposure to high altitude and coronary ectasia.

## RESUMEN

**Objetivo.:**

Evaluar la asociación entre la exposición crónica a gran altura y la presencia de ectasia coronaria (EC) en pacientes con infarto de miocardio con elevación del segmento ST (IAMCEST) tratados en un hospital de referencia de alta especialidad cardiovascular en Perú.

**Materiales y métodos.:**

Estudio retrospectivo de casos y controles emparejados. Los casos fueron pacientes con EC y los controles sin EC. Se evaluó la relación entre EC y exposición crónica a la altura, teniendo en cuenta variables intervinientes como hipertensión arterial, diabetes *mellitus*, dislipidemia, tabaquismo y valores de hematocrito. Se excluyeron los pacientes con patologías inflamatorias crónicas, enfermedad pulmonar obstructiva crónica y revascularización previa. Se aplicó una regresión logística multivariable para obtener el valor OR y sus respectivos intervalos de confianza.

**Resultados.:**

Se estudiaron 18 casos y 18 controles, la mayoría hombres con una edad media de 65 años. El 36% de la población procedía de zonas de gran altitud, de ellos, el 76,9% presentaba ectasia coronaria en la arteria responsable del infarto. El valor medio de hematocrito era ligeramente superior en los nativos de altitud (46 ± 7% frente a 42 ± 5%, p=0,094). La regresión logística condicional multivariable no encontró una relación significativa entre la exposición a gran altitud y el riesgo de presentar EC (OR:6,03, IC95%: 0,30-118, p=0,236).

**Conclusiones.:**

En pacientes con IAMCEST no encontramos asociación entre la exposición crónica a gran altitud y la presencia de ectasia coronaria.

## Introduction

ST elevated myocardial infarction (STEMI) is a frequent cause of mortality and morbidity worldwide, and among its causes, some abnormalities of the coronary arteries, such as coronary ectasia (CE), are reported in up to 3% of cases ^1^^,^^2^.

It is assumed that myocardial infarction in patients with CE is more frequent than in patients without CE (26.8% vs. 19.1%) ^3^, and in one-third of CE cases that are discovered during acute coronary syndrome, the infarct-related artery (IRA) had CE ^4^.

Some data link the origin of CE to alterations in nitric oxide (NO) function, inflammation, and damage to the coronary collagen matrix ^5^. It is also known that people living at high altitudes have overexpression of NO, endothelial damage, and products that can alter the proper function of coronary arteries ^6^. Observations from daily practice in the study center have found more cases of CE in patients with STEMI who are referred from high-altitude places to the hospital for reperfusion treatment.

To date, no direct association has been described in the literature between chronic exposure to high altitudes and the development of CE complicated with STEMI. Given that a significant percentage of the population in our country lives in high-altitude cities and that cardiovascular disease represents the second leading cause of death (especially of ischemic etiology), the objective of this study is to evaluate the association between chronic exposure to an altitude above 2500 meters above sea level (masl) and the presence of CE in patients with STEMI.

## Materials and methods

### Design and population

A matched case-control study was conducted, matching cases and controls by age and sex, using the myocardial infarction database of the study center. It included patients older than 18 years with STEMI treated at the National Cardiovascular Institute INCOR in Lima, Peru, between 2016 and 2022.

Cases were patients with STEMI who had CE on coronary angiography of the IRA, while controls were patients with STEMI without CE on angiography of the IRA.

Patients with a history of myocardial infarction and/or myocardial revascularization, rheumatological or chronic obstructive pulmonary disease, native from sea level or an altitude below 2500 masl regardless of the number of years lived at high altitude, native of high altitude living at sea level or below 2500 masl, medical records that did not indicate place of origin and birthplace, pregnant women, and undetermined culprit artery were excluded from the study.

### Sample design

As there was no data in the literature, a pilot study was conducted to determine the proportion of patients with STEMI exposed to high altitudes during 2016 and treated in the study center. Out of 191 STEMI cases, 15 patients (7.9%) were from high-altitude cities. A sample size estimation was performed for the pilot study with a 99% confidence interval (CI), resulting in a sample size of 56 cases with STEMI. It was found that 40% of patients exposed to high altitudes and 3% of those not exposed to high altitudes had CE. This resulted in an odds ratio (OR) of 16.3 for the presence of CE in those exposed to high altitudes.

Using these data from the pilot study, the sample size calculation for a matched paired case-control study was performed. With a 95% confidence level, 80% power, a case-control ratio of 1:1, the percentage of controls exposed to high altitudes according to the pilot study (5.7%), and the percentage of cases exposed to high altitudes of 50%, a minimum sample size of 18 pairs was obtained, making a total of 36 patients with STEMI for the study.

### Study variables

The dependent variable was the presence of CE in the IRA, evidenced during coronary angiography. The independent variable was chronic exposure to high altitudes, defined as the habitual residence of the patient at an altitude greater than or equal to 2500 masl. To determine whether a case or control met the criterion of chronic exposure to high altitude, data from medical history were used first (indicating the patient’s habitual residence at high altitude). Additionally, the patient’s national identification document’s location data and the presence of prior medical visits to the healthcare center in the high-altitude location were checked to corroborate these details.

Intervening variables included a history of hypertension, type 2 diabetes mellitus, dyslipidemia (a history of diagnosed hypercholesterolemia, hypertriglyceridemia, or both, or elevated LDL cholesterol and/or triglycerides values on admission to the hospital), smoking (current smoking history), and hematocrit at the time of hospital admission.

### Data collection procedures and techniques

The data were obtained from the medical records of patients with STEMI treated at the study center. The images of coronary angiography for cases described as having CE were evaluated by two interventional cardiologists to determine whether the diagnosis was correct. Both cardiologists made their diagnoses individually, and a third opinion was sought in case of disagreement. Controls were randomly selected from the STEMI database of the study center and matched to the cases in a 1:1 ratio by sex and age (+/- 5 years).

### Statistical analysis

Variables were expressed as frequencies and percentages for categorical variables and as the means or medians with their respective dispersion measures for continuous variables, depending on the distribution. Measures of association between categorical variables were calculated using the chi-squared test; for numerical variables, the t-test was used for normally distributed data.

Hypothesis testing was performed by determining the OR between exposure to high altitude and CE. Finally, because the case-control study was matched by age and sex, a conditional logistic regression analysis was conducted to determine the impact of other confounding variables. All statistical procedures were performed using Stata® software version 17.

### Ethical considerations

The study protocol was approved by the Ethics Committee of the Universidad Peruana Cayetano Heredia and the Ethics Committee of the National Cardiovascular Institute-INCOR. As it was a retrospective study based on medical record data, informed consent was not needed. No patient-identifying data were collected, and all information was for the exclusive use of the researcher. No patient was involved in the design or conduction of this research.

## Results

**Figure 1:**
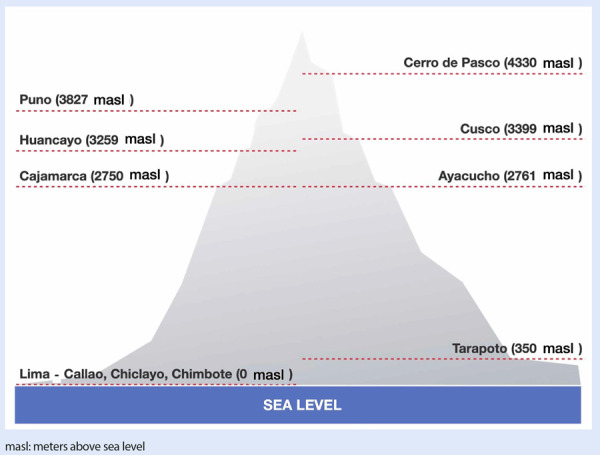
Altitude above sea level of the patient´s hometown

Over six years (2016-2022), 18 cases of STEMI and CE were found and were compared to 18 patients without CE. The overall average age of the population was 65 ± 9 years; 89% were males, 58% had hypertension, 22% had diabetes, 72% had dyslipidemia, and 19.4% were smokers. Thirteen patients came from high-altitude locations (36.1%), including the cities of Cusco (5 cases), Cajamarca (2 cases), Huancayo (2 cases), Puno (2 cases), Ayacucho (1 case), and Cerro de Pasco (1 case). Twenty-three patients (63.9%) came from sea-level locations (Lima, Callao, Chiclayo, Chimbote), and one patient from the city of Tarapoto (350 masl), **(**Figure 1**)**.

masl: meters above sea level

The characteristics of the study population are presented in Table 1. Among patients exposed to high altitudes, 76.9% had CE of the IRA, while in those not exposed to high altitudes, the finding of CE was 34.7% (p=0.035).

**Table 1:** Clinical characteristics of cases and controls.

Variable	Cases with CE	Controls without CE	P value
Age (years)*	65.7 ± 9.8	65.5 ± 9.6	0.946
Hematocrit*	46.2 ± 7.3	42.5 ± 5.3	0.094
Male sex	16 (88.8%)	16 (88.8%)	1.000
Patient from high altitude (> 2500 masl)	10 (55.6%)	3 (16.7%)	0.035
Hypertension	12 (66.7%)	9 (50%)	0.500
Diabetes mellitus type 2	1 (5.6%)	7 (38.8%)	0.041
Dyslipidemia	13 (72.2%)	13 (72.2%)	1.000
Smoking	5 (27.8%)	2 (11.1%)	0.402

* Mean and standard deviation

CE: Coronary Ectasia

masl: meters above sea level

**Figure 2:**
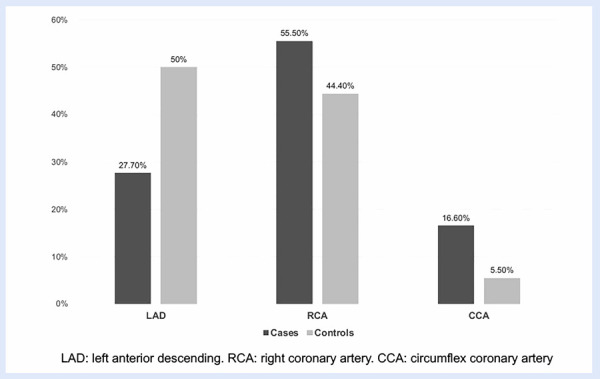
Infarct-related coronary artery in cases and controls.

The average hematocrit was slightly higher in cases with CE than in controls, with no significant difference (46.2% versus 42.5%; p=0.094). Similarly, the average hematocrit values were higher in high-altitude natives (49.7% ± 6.6 and 41.3% ± 4.3, p=0.0001). In the CE cases, the IRA most affected was the right coronary artery (55.6%), while in controls, it was the left anterior descending artery (50%), **(**Figure 2**)**.

The bivariate and multivariate conditional logistic regression analysis revealed that high-altitude residents with STEMI had six times the risk of having CE of the IRA, but this relationship was not statistically significant **(**Table 2**)**.

**Table 2:** Bivariate and multivariate conditional logistic regression analysis.

Variable	Bivariate analysis		Multivariate analysis	
	OR (CI 95%)	P value	OR (CI 95%)	P value
Patient from high altitude (> 2500 masl)	3.3 (0.9 - 12.1)	0.067	6.03 (0.30- 118)	0.236
Hypertension	2 (0.5 - 7.9)	0.327	0.6 (0.04 - 8.1)	0.704
Dyslipidemia	1 (0.2 - 4.9)	1.000	0.9 (0.12 - 8.1)	0.994
Smoking	2.5 (0.4 - 12.8)	0.273	22.3 (0.7 - 667)	0.073
Hematocrit	1.1 (0.9-1.2)	0.127	1.14 (0.9 - 1.4)	0.290

masl: meters above sea level

## Discussion

In this retrospective matched case-control study of STEMI patients treated at a national reference center in Peru, no association was found between exposure to high altitudes and CE when adjusted for other risk factors, such as hypertension, dyslipidemia, smoking, and hematocrit value.

CE is a diffuse abnormal dilatation of the epicardial coronary artery greater than 1.5 times the adjacent coronary segment and exceeds more than one-third of its length, is diagnosed by coronary angiography or coronary CT angiography ^7^ and can be found in 2.7% of coronary angiographies, is more common in young men and coexists with coronary atherosclerotic disease in 87% of cases ^8^. The prevalence of CE in patients with STEMI is 3 to 3.2%, and it has been associated with major adverse events during follow-up ^1^^,^^2^. Furthermore, it is assumed that myocardial infarction in patients with CE is more frequent than in patients without CE (26.8% vs. 19.1%) ^3^, and in one-third of CE cases discovered during acute coronary syndromes, the IRA has CE ^4^.

CE etiopathology is related to some nitric oxide-derived products that chronically stimulate the vascular endothelium, cause coronary dysfunction, and develop CE through the activation of matrix metalloproteinases, which are enzymes that degrade the extracellular matrix of the vascular wall ^5^. Other clinical conditions related to CE are classic endothelial dysfunction, rheumatological diseases, Kawasaki disease, familial hypercholesterolemia, Chlamydia pneumoniae infections, cocaine addiction, sickle cell disease ^5^^,^^7^^,^^9^, genetic predisposition ^10^, elevated triglyceride levels, LDL/HDL ratio, and other known coronary risk factors (hypertension, diabetes mellitus, smoking) ^11^^,^^12^.

Studies in high-altitude residents (living above 2500 meters above sea level [masl]) ^13^ have found overexpression of NO with abnormal production of endothelial damage-related products ^6^. Hypobaric hypoxia, which is responsible for the development and progression of acute and chronic mountain sickness ^14^^,^^15^, occurs in almost 10% of the population in the Andes above 2500 masl ^16^ and has been linked to inflammatory and oxidative response, increasing the levels of oxidative-inflammatory-nitrosative (OXINOS), which has been associated with endothelial dysfunction and structural damage to the blood vessels ^6^.

In addition, other classic coronary disease risk factors could be the origin of endothelial dysfunction in this population. For instance, arterial hypertension may be higher as altitude increases (Tibetan natives) or lower as altitude increases above sea level (Andean natives) ^17^. Regarding dyslipidemia, there is a higher prevalence of hypertriglyceridemia and reduced HDL cholesterol in Tibetan natives, although findings are controversial and strongly associated with lifestyle and socioeconomic factors ^13^. For type 2 diabetes mellitus (DM2), an inverse association with altitude has been reported in males ^18^, and a cross-sectional study in Peru found an inverse association between altitudes above 2500 masl and the 10-year Framingham risk score, particularly in terms of the presence of diabetes mellitus ^19^.

Globally, approximately 81 million people live at high altitudes, representing 1.07% of the world’s population. In Peru, approximately 8 million people live above 2500 masl, constituting 25% of the Peruvian population ^20^. There are limited data on the prevalence of CE in Peru. A recent study found a prevalence of CE of 1.2% of total catheterizations over eight years (91 cases), with 15% of these (13 cases) associated with STEMI ^21^. The same authors reported a higher frequency of CE associated with acute coronary syndromes in patients from cities above 2500 masl, and the right coronary artery was the most affected coronary artery, as in this report.

Our population is similar to what Wang reported, where CE associated with STEMI was higher in males in their sixth decade of life (74% of males with an average age of 62 years) ^1^. While the study found that hematocrit was slightly higher in high-altitude residents, only one patient met the criteria for chronic mountain sickness (hematocrit of 63% in a patient from the city of Puno). Ninety percent of high-altitude patients had a hematocrit of less than 60%. This elevation of hematocrit could be a link between exposure to high altitudes and the development of CE, emphasizing the importance of studying other factors related to high-altitude exposure (inflammation, hyperviscosity, endothelial dysfunction, etc.) that could influence the occurrence of CE.

Despite the physiological and pathophysiological changes found in high-altitude residents, this study failed to establish a relationship between these changes and the occurrence of CE, possibly due to the study’s design (matched prevalent case-control), the small sample size, and the use of data from medical records not collected by the researcher.

The importance of further studying the relationship between CE and exposure to high altitudes lies in the potential for implementing special treatment measures for STEMI patients from high altitudes. For example, fibrinolysis (within the appropriate time frame) before invasive treatment to reduce the typical thrombotic burden in CE patients or the use of coronary thrombus aspiration as a standard measure during catheterization. While these measures could be effective, more studies are needed to demonstrate their utility in STEMI patients with CE. Currently, there are reports of increased use of thrombectomy and a lower rate of stent implantation in this context ^1^.

There is also controversy regarding anticoagulation in the group of postmyocardial infarction patients with CE. Multivariate analysis revealed that the risk of major cardiac events (MCE) is higher in patients with myocardial infarction due to CE (HR 4.94, 95% CI 2.36-10.4, p<0.001) during 4-year follow-up and that with appropriate anticoagulation use (warfarin with optimal time in therapeutic range >=60%), this risk of MCE significantly decreases ^2^. Currently, there is an ongoing study in Latin America that will attempt to determine the best anticoagulation-antiplatelet strategy for these patients ^22^.

The study has limitations inherent to a prevalent case-control (reporting bias) and a small sample size, which is related to the low prevalence of the disease in the population. Therefore, a definitive association between exposure to high altitudes and the presence of CE in STEMI patients cannot be ruled out, which leads to the possibility that a type II error may have occurred during the study (rejecting the alternate hypothesis when it is true).

Since this is a retrospective study, other factors related to the presence of CE mentioned in the literature (including laboratory values) were not studied due to the absence of complete data in the medical records. The results may only apply to the STEMI population because, for ease of conducting a coronary angiography (the gold standard) in these patients, this diagnostic method was chosen over coronary computed to angio-tomography for patients with chronic coronary syndrome, as it is not available in many high-altitude locations from which our patients originated.

In conclusion, in this study of STEMI patients, there was no association between chronic exposure to high altitudes and the presence of CE of the IRA. I suggest implementing a nationwide incident case-control study of CE in the entire context of coronary artery disease (stable and unstable angina and infarction) and not only in STEMI. In addition to being able to collect important data to ensure chronic exposure to high altitude (including from previous generations of the patient), relevant laboratory and clinical data could also be obtained to better characterize the association between exposure to high altitude and the development of CE.

## References

[1] Wang X, Montero-Cabezas JM, Mandurino-Mirizzi A, Hirasawa K, Ajmone Marsan N, Knuuti J (2021). Prevalence and Long-term Outcomes of Patients with Coronary Artery Ectasia Presenting with Acute Myocardial Infarction. Am J Cardiol.

[2] Doi T, Kataoka Y, Noguchi T, Shibata T, Nakashima T, Kawakami S (2017). Coronary artery ectasia predicts future cardiac events in patients with acute myocardial infarction. Arterioscler Thromb Vasc Biol.

[3] Sheng Q, Zhao H, Wu S, Liu R (2020). Underlying factors relating to acute myocardial infarction for coronary artery ectasia patients. Medicine (Baltimore).

[4] Valente S, Lazzeri C, Giglioli C, Sani F, Romano SM, Margheri M (2007). Clinical expression of coronary artery ectasia. J Cardiovasc Med (Hagerstown).

[5] Manginas A, Cokkinos D V (2006). Coronary artery ectasias imaging, functional assessment and clinical implications. Eur Heart J.

[6] Tymko MM, Tremblay JC, Bailey DM, Green DJ, Ainslie PN, Petersen O (2019). The impact of hypoxemia on vascular function in lowlanders and high altitude indigenous populations. J Physiol.

[7] Ozturk S, Yetkin E, Waltenberger J (2018). Molecular and cellular insights into the pathogenesis of coronary artery ectasia. Cardiovasc Pathol.

[8] Giannoglou G, Antoniadis A, Chatzizisis Y, Damvopoulou E, Parcharidis G, Louridas G (2006). Prevalence of ectasia in human coronary arteries in patients in northern Greece referred for coronary angiography. Am J Cardiol.

[9] Gurlek A, Esenboga K, Ozcan OU, Cicek OF, Ayral PA, Kavas GO (2016). Serum nitric oxide levels in patients with coronary artery ectasia. Anatol J Cardiol.

[10] Yalcin AA, Akturk IF, Celik O, Erturk M, Hancer VS, Yalcin B (2014). Coronary Artery Ectasia Is Associated with the c 894G&gt;T (Glu298Asp) Polymorphism of the Endothelial Nitric Oxide Synthase Gene. Tohoku J Exp Med.

[11] Qin Y, Tang C, Ma C, Yan G (2019). Risk factors for coronary artery ectasia and the relationship between hyperlipidemia and coronary artery ectasia. Coron Artery Dis.

[12] Dastgir N, Masood A, Muqeet A, Khan Niazi GZ (2020). Frequency of risk factors in patients of acute coronary syndrome due to coronary ectasia. Asian Cardiovasc Thorac Ann.

[13] Bigham AW (2016). Genetics Of Human Origin and Evolution High-Altitude Adaptations. Curr Opin Genet Dev.

[14] Mallet RT, Burtscher J, Richalet JP, Millet GP, Burtscher M (2021). Impact of High Altitude on Cardiovascular Health Current Perspectives. Vasc Health Risk Manag.

[15] Gazal S, Espinoza JR, Austerlitz F, Marchant D, Macarlupu JL, Rodríguez J (2019). The Genetic Architecture of Chronic Mountain Sickness in Peru. Front Genet.

[16] Leon-Velarde F, Arregui A, Vargas M, Huicho L, Acosta R (1994). Chronic mountain sickness and chronic lower respiratory tract disorders. Chest.

[17] Aryal N, Weatherall M, Bhatta YKD, Mann S (2016). Blood Pressure and Hypertension in Adults Permanently Living at High Altitude A Systematic Review and Meta-Analysis. High Alt Med Biol.

[18] Woolcott OO, Castillo OA, Gutierrez C, Elashoff RM, Stefanovski D, Bergman RN (2014). Inverse association between diabetes and altitude A cross-sectional study in the adult population of the United States. Obesity.

[19] Hernández-Vásquez A, Vargas-Fernández R, Chacón-Diaz M (2022). Association between Altitude and the Framingham Risk Score A Cross-Sectional Study in the Peruvian Adult Population. Int J Environ Res Public Heal.

[20] Tremblay JC, Ainslie PN (2021). Global and country-level estimates of human population at high altitude. Proc Natl Acad Sci U S A.

[21] Rodríguez D, Rafael-Horna E, Quiroz J, Lévano-Pachas G, Meneses G (2022). Características clínicas y angiográficas de pacientes con ectasia coronaria en un hospital de referencia. Arch Peru Cardiol Cir Cardiovasc.

[22] Araiza-Garaygordobil D, Gopar-Nieto R, Sierra-Lara Martínez D, Belderrain-Morales N, Sarabia-Chao V, Alfaro-Ponce DL (2022). Dual Antiplatelet Therapy Versus Antiplatelet Monotherapy Plus Oral Anticoagulation in Patients with Acute Coronary Syndrome and Coronary Artery Ectasia Design and Rationale of OVER-TIME Randomized Clinical Trial. High Blood Press Cardiovasc Prev.

